# Inflammatory myofibroblastic tumor of the larynx: a case report

**DOI:** 10.3389/fonc.2026.1898366

**Published:** 2026-08-10

**Authors:** Marlene Reithofer, Lejla Pesto, Tobias Hütten, Anja Jurejevcic, Martina Rößmann-Tsybrovskyy, Hannaleena Tervonen, Dietmar Thurnher

**Affiliations:** 1 Department of Otorhinolaryngology, Medical University of Graz, Graz, Austria; 2 Diagnostic and Research Institute of Pathology, Medical University of Graz, Graz, Austria

**Keywords:** ALK fusion, case report, inflammatory myofibroblastic tumor, laryngeal tumor, microlaryngoscopy

## Abstract

**Background:**

Inflammatory myofibroblastic tumor (IMT) is a rare mesenchymal neoplasm characterized by myofibroblastic spindle cells and inflammatory infiltrates.

**Case summary:**

We report the case of a 49-year-old female presenting with progressive hoarseness and a right vocal fold mass. Microlaryngoscopy with partial resection was performed without preoperative imaging. Histopathological, immunohistochemical, and molecular analyses were conducted. Histopathological and molecular analyses showed a myofibroblastic spindle cell proliferation with a TIMP3::ALK gene fusion, confirming the diagnosis of IMT. Postoperative recovery was uneventful, and follow-up examinations demonstrated complete remission.

**Conclusion:**

Laryngeal inflammatory myofibroblastic tumor (IMT) is a very rare condition, particularly in adults. Complete surgical excision can lead to full remission, but regular follow-up is important due to the risk of recurrence. Immunohistochemical and molecular analyses, especially ALK testing, are essential for accurate diagnosis and evaluation regarding targeted therapy. Further studies are needed to establish clear recommendations for follow-up and treatment.

## Introduction

An Inflammatory Myofibroblastic Tumor (IMT) is an extremely rare neoplasm characterized by a proliferation of myofibroblastic and fibroblastic spindle cells, typically along with a prominent inflammatory infiltrate composed of plasma cells, lymphocytes, and eosinophils (1).

It usually occurs in the lung, abdomen, pelvis, or retroperitoneum and in children or young adults. There are only a few cases reported where this tumor affected the larynx (2).

Head and neck IMTs have mostly been reported in adults, without a clear sex predilection.

This tumor is described as a borderline tumor which means it shows a high tendency for local recurrence following excision, while their metastatic potential remains low (3).

Molecularly, clonal cytogenetic rearrangements of the ALK gene at chromosome band 2p23 can be detected in approximately 50–60% of IMTs, particularly in children and young adults (3). These rearrangements result in fusion of the 3′ kinase domain of ALK with a variety of partner genes, such as TPM3, TPM4, CLTC, CARS, SEC31L1, PPFIBP1, ATIC, EML4, PRKAR1A, FN1, DCTN1, LMNA, TFG, and HNRNPA1. More fusion partners are continuously being discovered (4).

Complete surgical excision is the treatment of choice for IMT, while targeted therapies such as ALK or ROS1 inhibitors are effective options in advanced or unresectable cases (4).

## Case description

A 49-year-old female was referred to the Department of Phoniatrics at the Medical University of Graz by a private otolaryngologist with a suspected vocal fold mass. She reported progressive hoarseness up to aphonia for two months, accompanied by sore throat. Initially, she also experienced dyspnea. At the time of evaluation, however, no clinically relevant respiratory distress was present, and endoscopic examination showed no evidence of significant airway compromise. Therefore, no additional objective assessment of airway obstruction was performed.

The patient worked as an interpreter and was therefore exposed to significant occupational voice strain. Due to the hoarseness and aphonia she was unable to continue her professional activity. Before presentation to our department, she had received antibiotic therapy and inhalations without clinical improvement.

Her medical history was unremarkable, with no comorbidities. She has a 30 packyear smoking history. Alcohol consumption history was not obtained.

**Figure 1:**
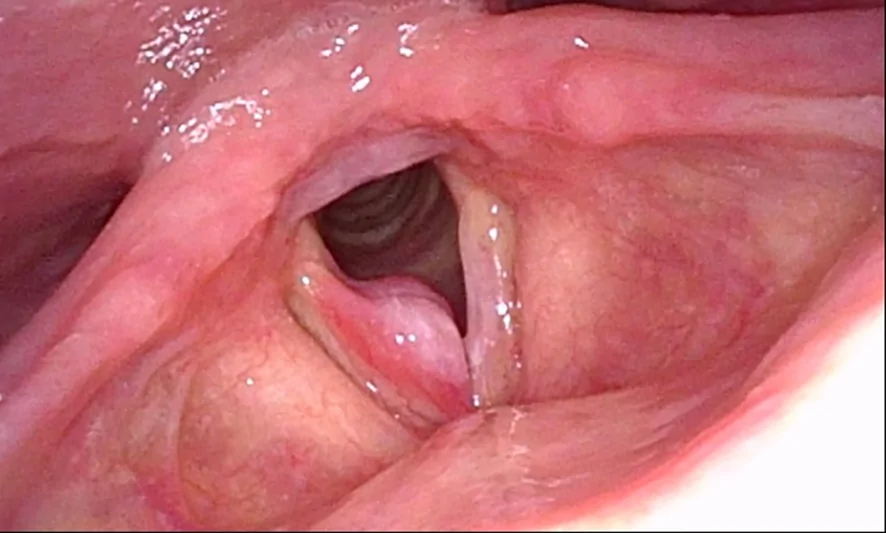
Clinical picture: tumor of the right vocal fold compatible with an IMT.

On physical examination, flexible laryngoscopy revealed a prominent, partially livid-colored tumor on the right vocal fold, causing incomplete glottic closure during phonation. Respiratory mobility of the vocal folds appeared normal (**Figure 1**). Based on the clinical presentation and endoscopic findings, the differential diagnosis included common benign vocal fold lesions such as a polyp, cyst, granuloma, or papilloma. Less common mesenchymal lesions and malignant neoplasms were also considered.

After initial presentation of the patient, a day-case microlaryngoscopy (MLX) under general anesthesia was scheduled. No preoperative imaging, such as MRI, was performed, as endoscopic examination showed a lesion limited to the vocal fold and there were no clinical signs of more extensive local involvement.

The surgery was performed 19 days later without any complications. During the microlaryngoscopy the IMT was resected using a rasp.

Due to severe postoperative pain, the patient was admitted to our ward for observation and initiation of antibiotic treatment Flexible endoscopic examination at that time revealed unremarkable postoperative wound conditions. She was discharged in good condition two days later. Home care instructions included a further treatment of antibiotics (amoxicillin/clavulanic acid) for one more week, anti-inflammatory pain medication for three days and antiseptic/anesthetic mouthwash. She was advised to rest her voice for several days. A follow-up appointment was arranged for two weeks postoperatively for clinical and histological evaluation.

**Figure 2:**
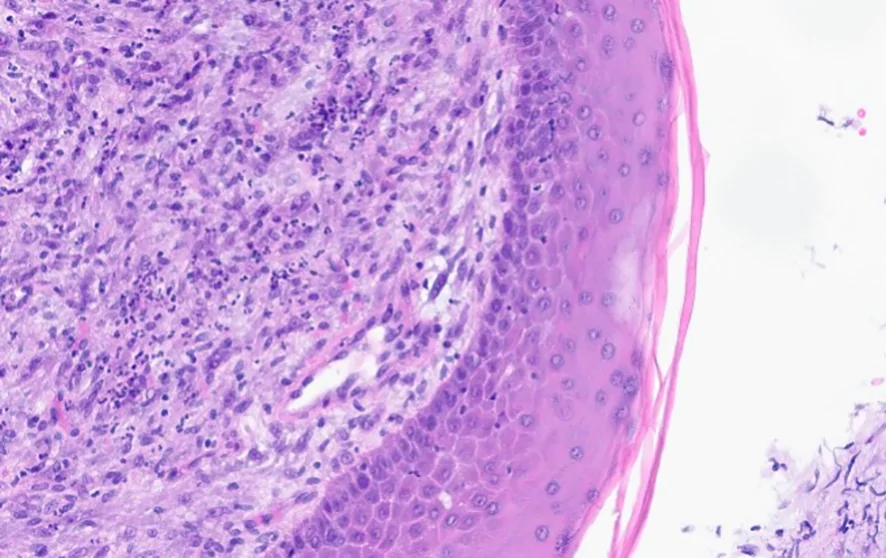
Histological picture (HE 20x): between the tumor cells there are scattered inflammatory cells.

The histological examination showed squamous mucosa with a spindle-cell, myofibroblastic proliferation with myxoid stroma and associated chronic inflammatory infiltrate (**Figure 2**).

**Figure 3:**
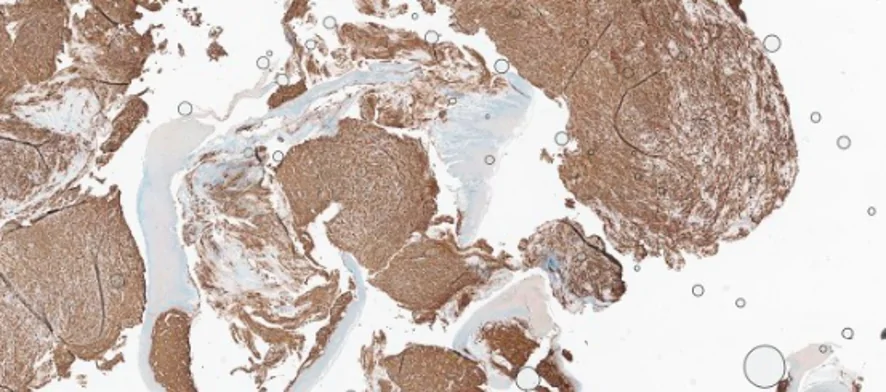
Tumor cells are typically positive for ALK by immunohistochemistry.

At the molecular level a “TIMP3::ALK” was identified (**Figure 3**). Integration of the histomorphological, immunohistochemical, and molecular findings was diagnostic of an IMT.

## Timeline

**Figure 4:**
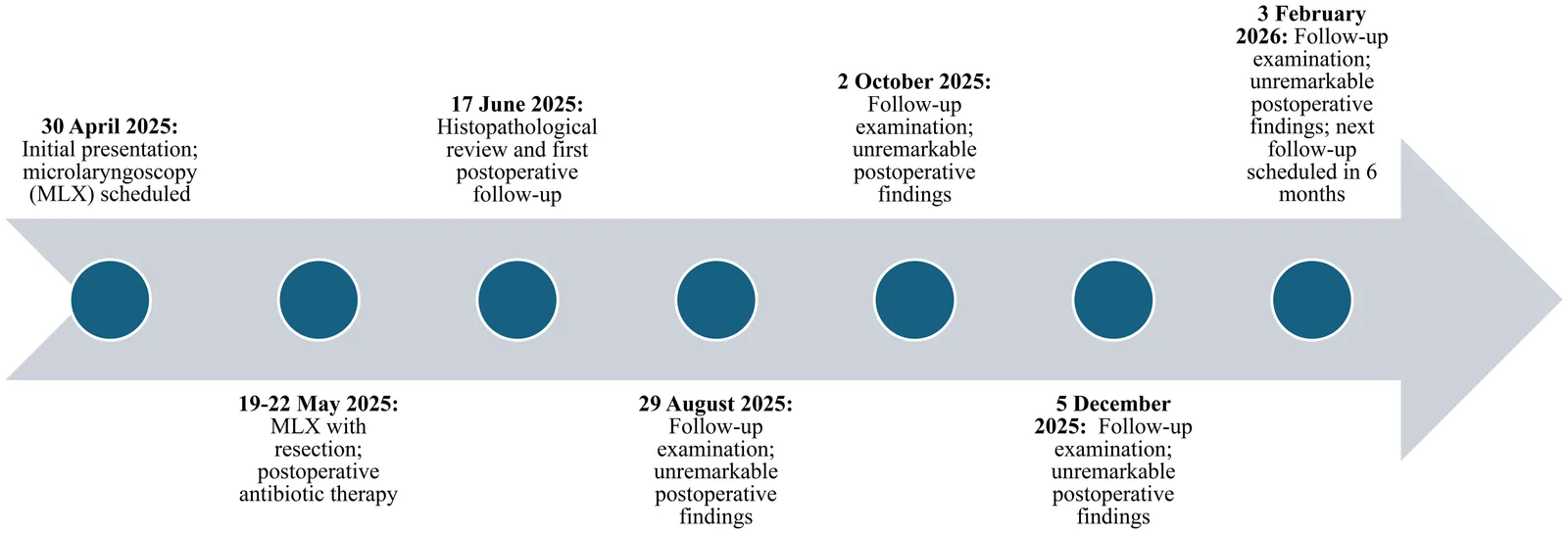
Timeline of clinical course: chronological overview of initial presentation, surgical intervention, histopathological diagnosis and follow-up examinations.

A timeline summarizing the clinical course is provided in **Figure 4**.

## Diagnostic assessment

Histopathological examination demonstrated a spindle-cell proliferation within a myxoid stroma and inflammatory infiltrates. Immunohistochemical analysis showed ALK positivity. Molecular testing identified a TIMP3::ALK fusion, confirming the diagnosis of an IMT.

## Therapeutic intervention

Complete microlaryngoscopic resection was performed under general anesthesia. No additional treatment was required.

## Follow-up and outcomes

Flexible endoscopy continued to show an unremarkable postoperative appearance over a 9-month-follow-up period. At follow-up, the patient reported marked improvement in voice quality and was satisfied with the postoperative outcome. She was able to resume her professional activities as an interpreter without limitations. Continued surveillance was planned, with the next follow-up examination scheduled six months after the most recent visit.

## Patient perspective

According to follow-up documentation, the patient reported marked improvement in voice quality and was satisfied with the treatment outcome.

## Discussion

Inflammatory myofibroblastic tumor (IMT) is a rare neoplasm that has been described in various anatomical sites, most commonly in the lung and abdomen. Involvement of the larynx, however, is extremely uncommon, with only a few cases reported to date (5).

To place our case into a current clinical context, we performed a focused PubMed search limited to adult laryngeal IMT cases published within the past ten years (2015-2025). Only a very small number of reports met these criteria, showing the rarity of this entity in adulthood. Clinical characteristics were extracted and summarized in **Table 1**.

**Table 1:** Reported cases of adult laryngeal inflammatory myofibroblastic tumors published within the last 10 years.

First author (ref.)	Year	Age (years)	Sex	Tumor location	Treatment
Izadi et al. (14)	2016	46	F	Right aryepiglottic fold and vallecula, Epiglottis	Laser resection
Zheng et al. (15)	2016	81	M	Right vocal fold	Surgical resection
Raggio et al (16)	2018	66	F	Epiglottis	Laser resection
Yorita et al (17)	2019	35	M	Left vocal fold	Surgical resection
Miralles et al (18)	2021	73	M	Left vocal fold	Laser resection
Sivesind et al (19)	2023	22	F	Right vocal fold	Surgical resection + steroid therapy
Ajmal et al (20)	2024	24	M	Right vocal fold	Surgical resection
Chen et al (21)	2025	39	M	Subglottis	Surgical resection + steroid therapy

Ref.: Reference; F: Female; M: Male.

In the series by Coffin et al., only three of 84 extrapulmonary IMTs involved the larynx, and all occurred in children (6). IMTs can occur in any part of the larynx, but most frequently involve the true vocal folds, followed by the subglottic region (7).

The etiology of laryngeal IMT remains uncertain. Possible contributing factors include prior trauma, infections, neoplastic processes, and immune reactions. Smoking may play a role, as approximately one quarter of reported laryngeal IMT patients were smokers. However, a causal association has not been established. Although Epstein–Barr Virus (EBV) has been linked to IMTs in other anatomical sites, no such association has been demonstrated in laryngeal cases (8).

Histologically, IMT must be differentiated from various morphologic mimics, such as Inflammatory Pseudotumor (IPT), IgG4-related sclerosing disease (IGSD), pseudosarcomatous myofibroblastic proliferation (PMP), inflammatory fibroid polyp (IFP), nodular fasciitis (NF) and many more (4).

Cook et al. demonstrated that approximately 60% of IMTs show ALK positivity on immunohistochemistry, whereas other morphologic mimics such as nodular fasciitis, desmoid fibromatosis, or gastrointestinal stromal tumors remain ALK negative (9). Immunohistochemical analysis demonstrated strong positivity for ALK and focal positivity for SMA. Staining for desmin, SOX10, S100, AE1/AE3, and CD34 was negative. These findings supported the diagnosis of IMT and helped exclude other spindle cell lesions in the differential diagnosis.

The identification of a TIMP3::ALK fusion is a noteworthy finding, as TIMP3 represents an uncommon ALK fusion partner in IMT. This further shows the molecular heterogeneity of ALK-rearranged tumors and highlights the importance of molecular testing in establishing the diagnosis. ALK positivity is particularly relevant from a therapeutic perspective as it identifies patients who may benefit from targeted therapy. The study by Butrynski et al. showed that around half of IMTs contained ALK fusion genes, making them responsive to targeted therapy with ALK inhibitors such as crizotinib. Although resistance to crizotinib can develop after several months, newer-generation ALK inhibitors and combination regimes are being explored to improve long-term outcomes (10). Given the diagnostic and potential therapeutic relevance of ALK rearrangements, molecular testing should be strongly considered in all histologically suspected IMTs.

The diagnosis of rare head and neck neoplasms can be challenging and may require expert pathological evaluation as well as additional diagnostic tests. Filippini et al. showed that the diagnosis of rare head and neck tumors may change after expert pathological review, emphasizing the importance of detailed histopathological and molecular analyses in rare tumors such as laryngeal IMT (11).

Another challenge in managing IMTs is the potential for local recurrence, even after complete surgical resection. The recurrence rate of laryngeal IMTs ranges from 8% to 18%, typically occurring between 2 and 12 months after initial treatment. Contributing factors for recurrence may include incomplete surgical excision (7). Therefore, regular postoperative follow-up is recommended. However, due to the rarity of laryngeal IMT, surveillance strategies are largely based on case reports and small case series, and clear follow-up recommendations have not yet been established. Similar challenges have been described for other rare laryngeal neoplasms. In a recent systematic review of laryngeal mucoepidermoid carcinoma, Chiari et al. highlighted the importance of long-term surveillance because of the risk of local, regional, and distant recurrence (12).

In the study by Gao et al. the author evaluated the characteristic imaging features of IMTs in this region. It showed that although CT and MRI help assess the extent and anatomical relationships of the lesion, the imaging findings are generally nonspecific and cannot reliably distinguish IMT from malignant tumors (13). In this case, no preoperative imaging was performed.

Our case illustrates the diagnostic and therapeutic challenges and highlights the importance of considering IMT in the differential diagnosis of vocal fold masses. Increasing awareness of this tumor is essential for distinguishing it from other aggressive lesions. An accurate diagnosis of IMT is important to ensure appropriate patient management and follow-up, while at the same time avoiding unnecessary interventions and potential harm.

In the present case, complete surgical resection achieved full remission, which has been maintained up to the most recent follow-up. Nevertheless, targeted therapies are likely to gain importance in the future, and further studies are required to define the optimal management of this rare neoplasm. Most laryngeal IMTs reported in the literature occur in children, making this case in an adult patient particularly unusual and noteworthy.

## Conclusion

Laryngeal inflammatory myofibroblastic tumor (IMT) is an exceptionally rare entity, particularly in adult patients. This case highlights the importance of considering IMT in the differential diagnosis of atypical vocal fold masses presenting with persistent hoarseness and demonstrates that complete surgical excision can lead to full remission. Immunohistochemical and molecular analyses, especially ALK testing, play a crucial role in confirming the diagnosis and may open the door to targeted treatment in recurrent, unresectable, or metastatic ALK-positive IMTs. Given the risk of local recurrence, regular postoperative follow-up should be considered even after complete resection.

## Data Availability

The original contributions presented in the study are included in the article/supplementary material. Further inquiries can be directed to the corresponding author.
